# Supplementary figures and images for: Weevil Carbohydrate Intake Triggers Endosymbiont Proliferation: A Trade-Off between Host Benefit and Endosymbiont Burden
Source: mBio. 2023 Feb 13;14(2):e03333-22. doi: 10.1128/mbio.03333-22 (PMC10127669; doi:10.1128/mbio.03333-22)

# Insect life cycle with spontaneous emergence from grain

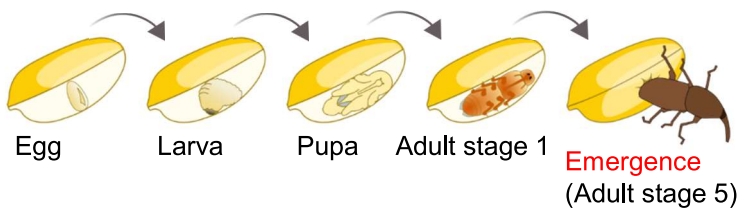

A

## Grain-reared insects

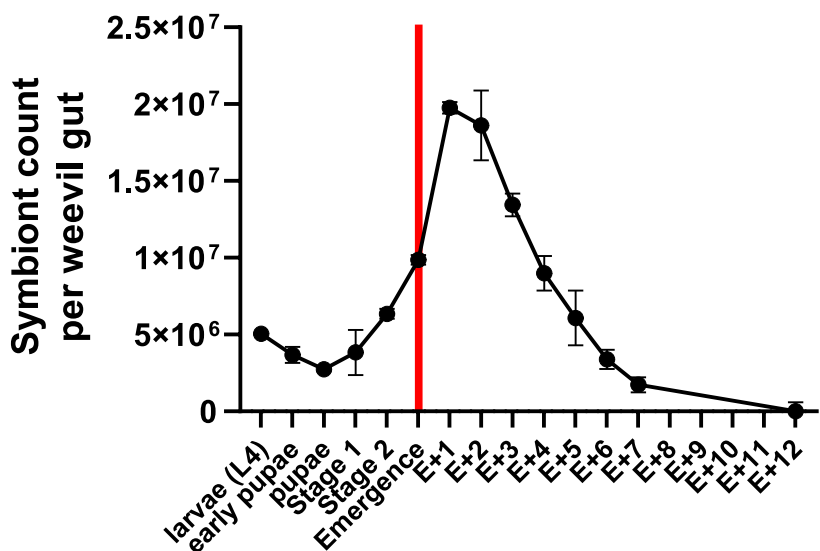

B

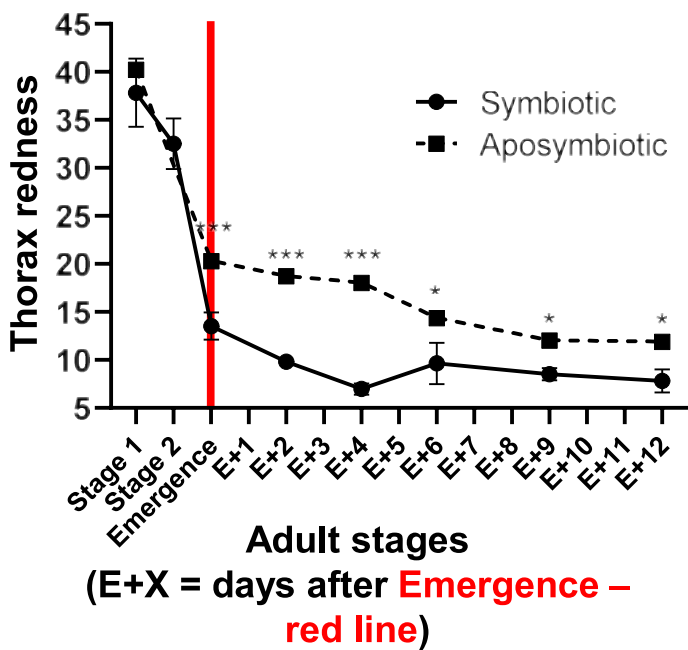

Supplement: FIG S1 [file mbio.03333-22-s0001.pdf]

A

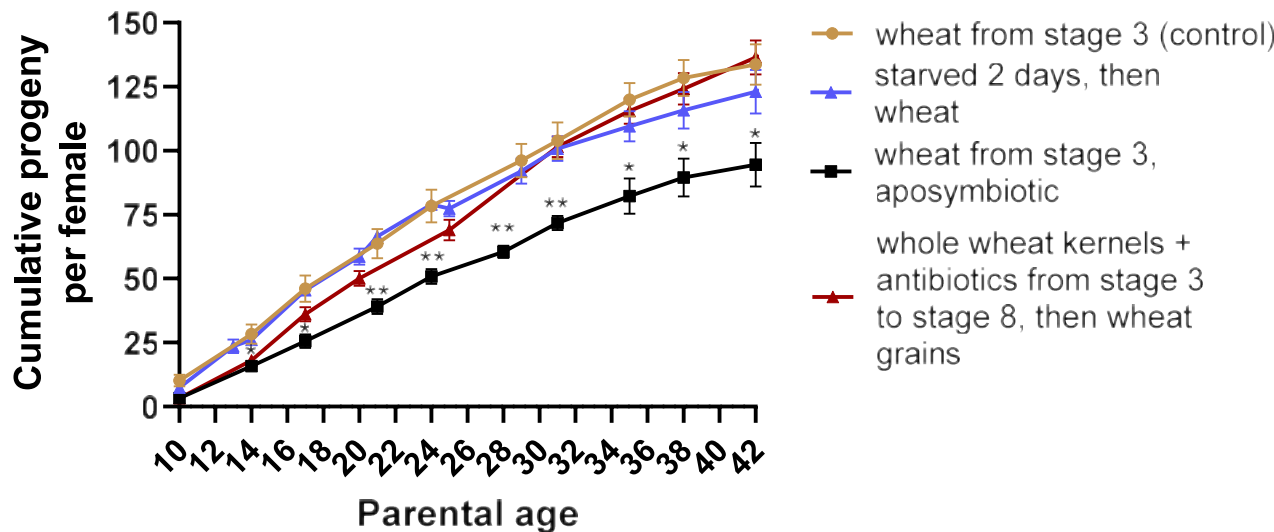

B

### Ovaries at stage 24

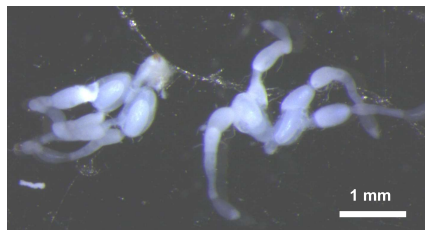

wheat from stage 3 (control)

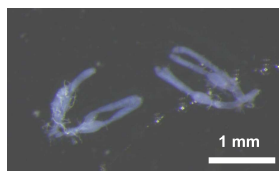

starch diet

Supplement: FIG S3 [file mbio.03333-22-s0003.pdf]

# Starvation effects on insects naturally emerged from wheat grains

A

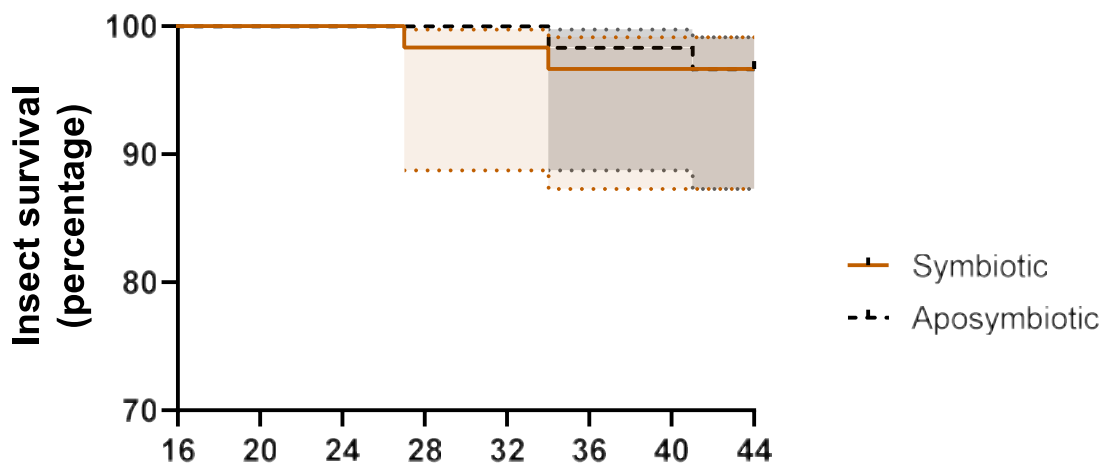

B

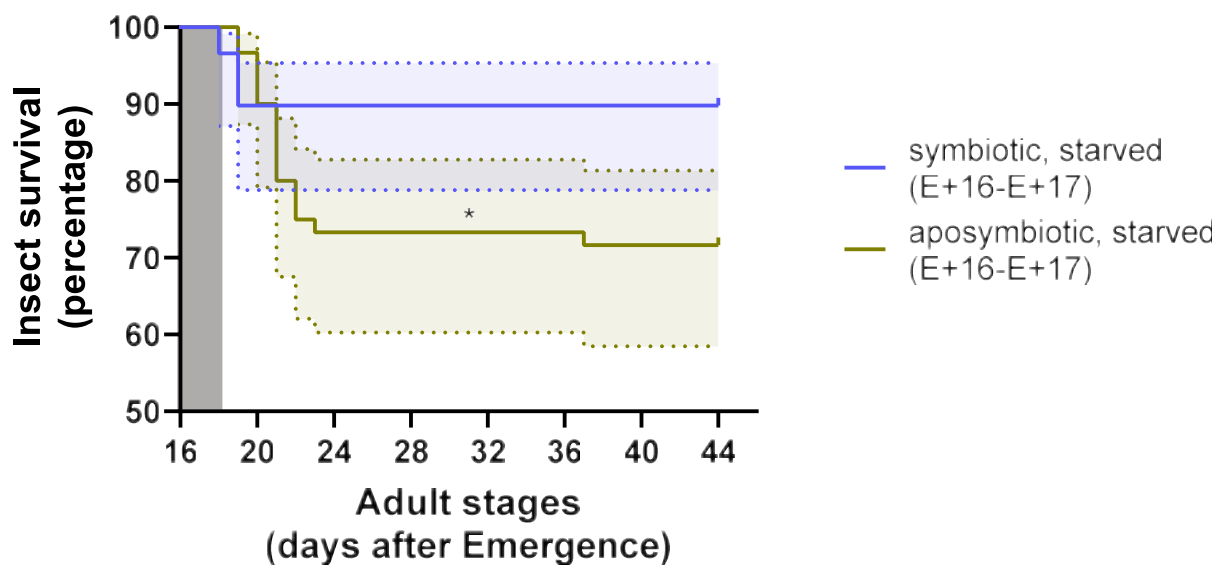

Supplement: FIG S4 [file mbio.03333-22-s0004.pdf]

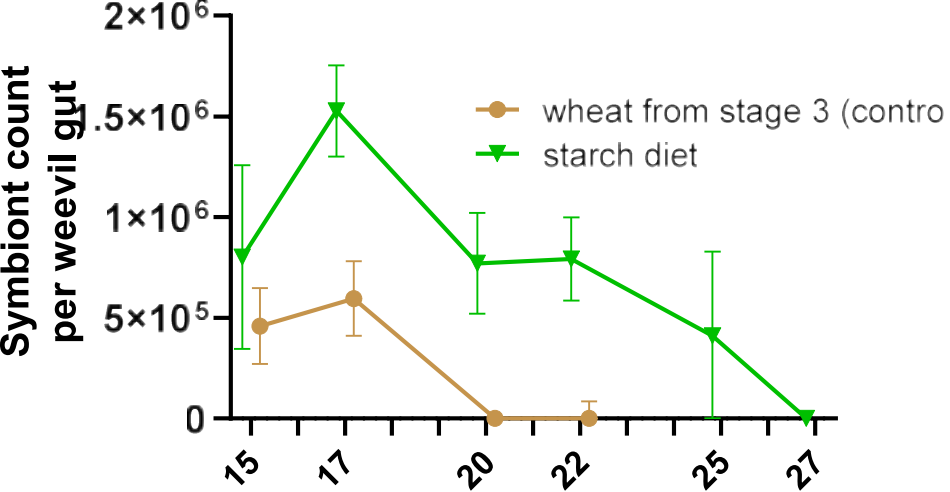

Supplement: FIG S5 [file mbio.03333-22-s0005.pdf]
