# Supplementary material for: Weevil Carbohydrate Intake Triggers Endosymbiont Proliferation: A Trade-Off between Host Benefit and Endosymbiont Burden
Source: mBio. 2023 Feb 13;14(2):e03333-22. doi: 10.1128/mbio.03333-22 (PMC10127669; doi:10.1128/mbio.03333-22)
Supplement: FIG S2 [file mbio.03333-22-s0002.pdf]

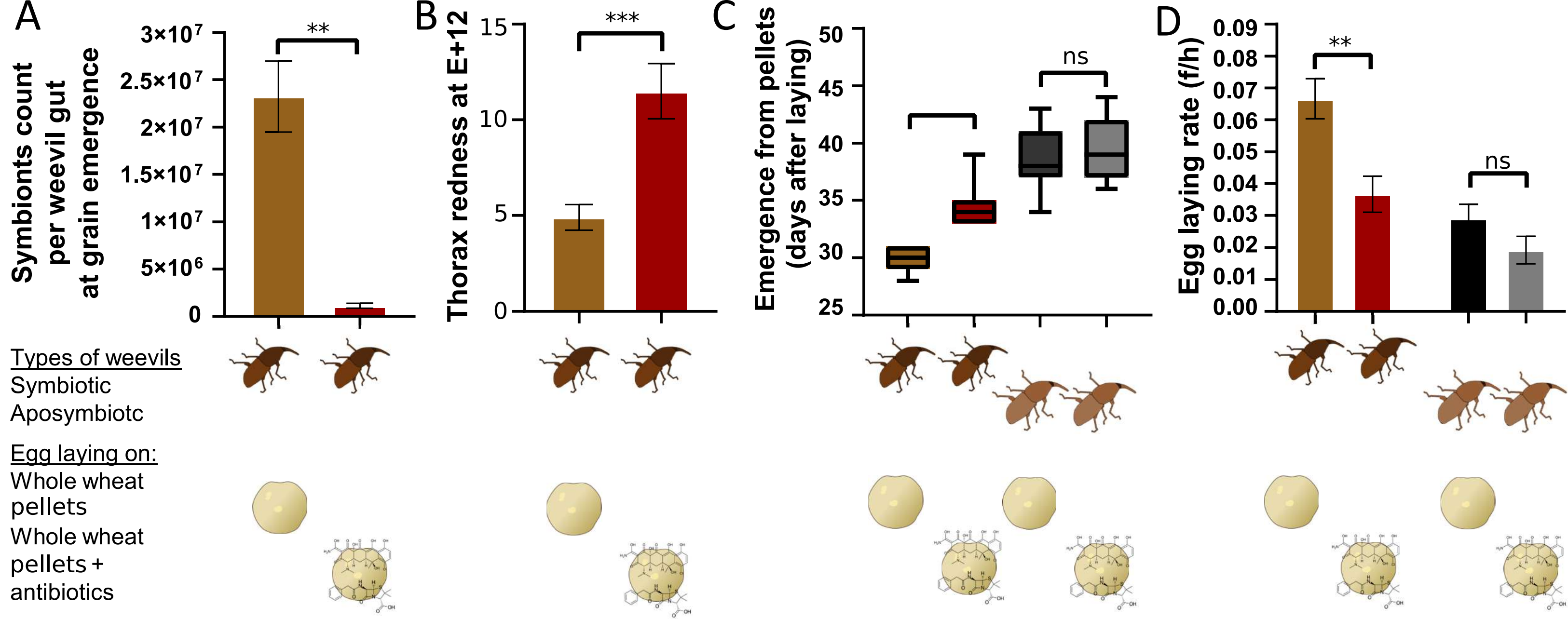

A
Symbionts count per weevil gut at grain emergence
3×10^7
2.5×10^7
2×10^7
1.5×10^7
1×10^7
5×10^6
0
**
B
Thorax redness at E+12
15
10
5
0
***
C
Emergence from pellets (days after laying)
50
45
40
35
30
25
ns
D
Egg laying rate (f/h)
0.09
0.08
0.07
0.06
0.05
0.04
0.03
0.02
0.01
0.00
**
ns
Types of weevils
Symbiotic
Aposymbiotc
Egg laying on:
Whole wheat pellets
Whole wheat pellets + antibiotics
