# Supplementary material for: Weevil Carbohydrate Intake Triggers Endosymbiont Proliferation: A Trade-Off between Host Benefit and Endosymbiont Burden
Source: mBio. 2023 Feb 13;14(2):e03333-22. doi: 10.1128/mbio.03333-22 (PMC10127669; doi:10.1128/mbio.03333-22)
Supplement: FIG S6 [file mbio.03333-22-s0006.pdf]

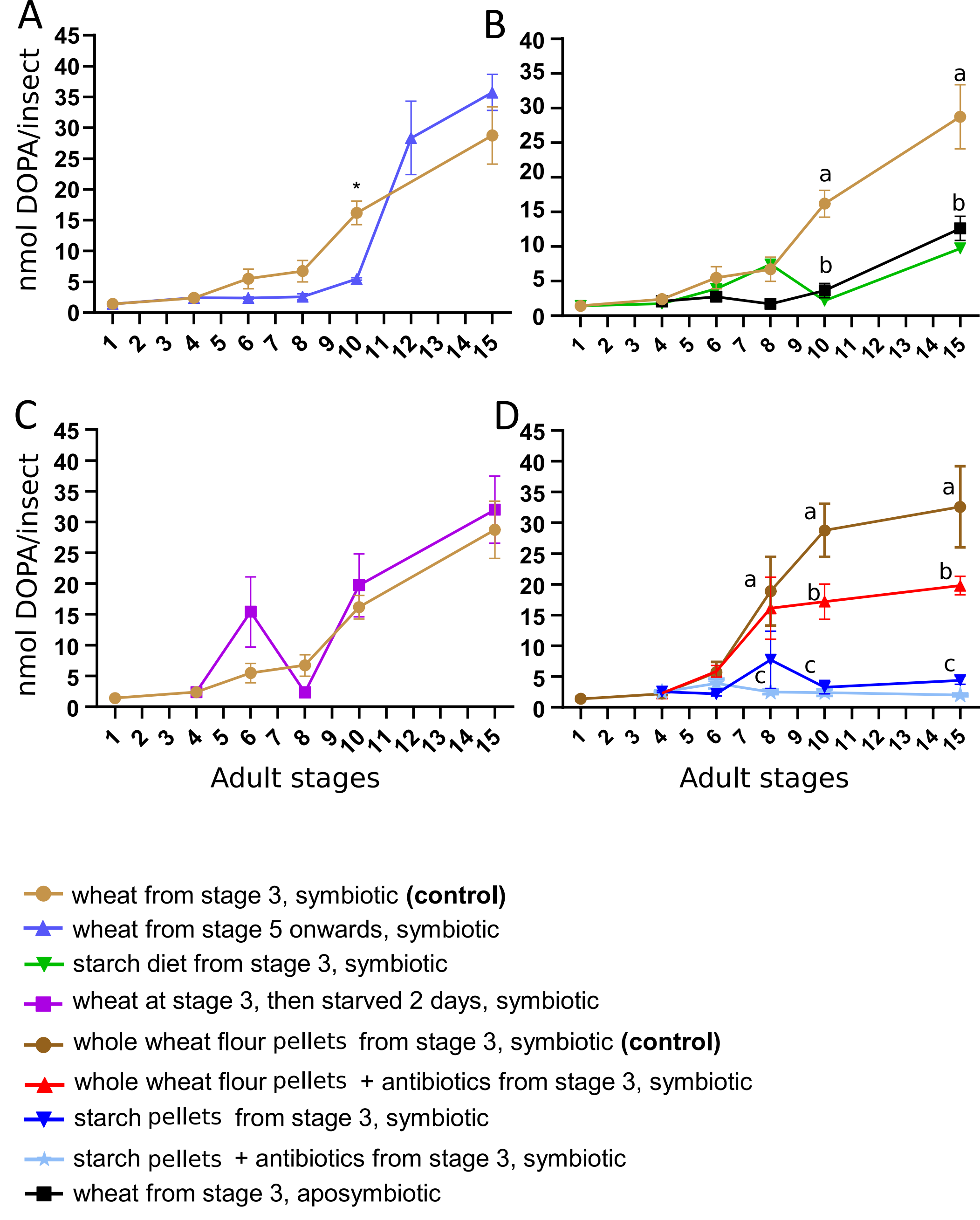

A
B
C
D
nmol DOPA/insect
Adult stages
*
a
b
c
wheat from stage 3, symbiotic **(control)**
wheat from stage 5 onwards, symbiotic
starch diet from stage 3, symbiotic
wheat at stage 3, then starved 2 days, symbiotic
whole wheat flour pellets from stage 3, symbiotic **(control)**
whole wheat flour pellets + antibiotics from stage 3, symbiotic
starch pellets from stage 3, symbiotic
starch pellets + antibiotics from stage 3, symbiotic
wheat from stage 3, aposymbiotic
